# Health Status of People Who Are and Are Not Experiencing Homelessness: Opportunities for Improvement

**DOI:** 10.3390/ijerph21101313

**Published:** 2024-10-01

**Authors:** Susan J. Gordon, Nicky Baker, Tania S. Marin, Margie Steffens

**Affiliations:** 1 College of Nursing and Health Sciences, Flinders University, Adelaide 5042, Australia; nicky.baker@flinders.edu.au (N.B.); tania.marin@flinders.edu.au (T.S.M.); 2 Adelaide Dental School, The University of Adelaide, Adelaide 5000, Australia; margie.steffens@adelaide.edu.au

**Keywords:** health status, homelessness, PEH, vulnerable population, public health

## Abstract

This study assessed the physical and psychological health parameters of adults experiencing homelessness to inform the development and delivery of health services by comparing with a housed population in the same South Australian city. Adults experiencing homelessness, known to existing support services, were invited to participate in a comprehensive assessment of their physical and mental health using questionnaires and objective assessments. Descriptive analyses using the percentage of participants failing to attain recommended published thresholds and accumulated health deficits for 16 health assessments were compared for the young group of people experiencing homelessness (18–40 years), the middle aged and older people experiencing homelessness (40–75 years), and a housed population of the middle aged and older people (40–75 years). Those experiencing homelessness had multiple and potentially inter-related health deficits compared with a population of people not experiencing homelessness in the same city. They were significantly less likely to meet healthy population norms for clinical frailty (*p* < 0.001), psychological distress (*p* < 0.001), grip strength (*p* < 0.001), lung function (*p* < 0.001), sleep quality (*p* < 0.001), and pelvic floor bother (*p* = 0.002). Significantly more accumulated health deficits were found for people experiencing homelessness when compared with the same ages for those who were not (mean 6.5 (SD 2.4) compared with 5.0 (SD 2.1)). This considerably increased for people experiencing homelessness aged less than 40 years (mean 8.7 (1.7)). Priorities for health service provision for people of different ages experiencing homelessness, when compared with housed community dwellers, have been described. The provision of targeted health assessments and service provision that specifically address healthcare needs among people experiencing homelessness are likely to have the biggest impacts across multiple health domains.

## 1. Introduction

Homelessness is a significant and complex biopsychosocial issue affecting persons of all ages and backgrounds [1,2,3]. This enduring public health concern is being tackled across the developed world with strategies ranging from emergency interventions, temporary short- or longer-term supportive housing schemes, to the provision of services for permanent supportive housing made possible by governments’ formal and informal policies and funding responses [1,4]. However, achieving success in reducing homelessness requires a sound understanding of the specific health needs of people experiencing homelessness (PEH), captured by contextually appropriate health assessments. While providing secure housing is an essential solution [5], improving underlying health conditions that impact the individual’s overall health is needed for those who will always have ongoing insecure housing arrangements. However, establishing priorities for health services and interventions that address the greatest need for specific homeless populations is lacking.

Adults experiencing homelessness are rarely positioned economically or socially to access adequate or timely healthcare. Consequently, their health is often crisis-managed by frequent unplanned attendances at after-hours medical care and hospital emergency departments, which can result in potentially avoidable ward admissions [5]. Compared with those not experiencing homelessness, PEH have a mortality rate up to six times higher and are found to die younger [6,7], made worse if experiencing infectious diseases, limitations in activities of daily living, and/or comorbidities [8]. Added to this are a range of environmental and psycho-social factors that limit PEH to access facilities for appropriate healthcare [9] or participate in society to the extent that they have some control over their environment [10].

In response, there have been a range of Australian policy recommendations proposed to address the health needs of PEH, including supply of coordinated and comprehensive healthcare services at established (safe) venues that are easily accessible and non-threatening and tailored to the health needs of end-users experiencing homelessness [11]. Almost 5 in every 1000 Australians (n = 122,494) were reported as experiencing homelessness in the 2021 Census [12], an increase of 5.2% from 2016. Although there are still overall more males experiencing homelessness in most Australian cities, females accounted for 81.7% of the 5.2% increase from 2016 to 2021—the largest new group of PEH. Additionally, 93,000 people were living in marginal housing (at risk of homelessness) [13]. However, ascertaining comparable numbers within and across countries is hindered by the lack of a global definition of homelessness [8], which challenges comprehensive global understanding of its prevalence and consequences [14].

In this paper, we compare the contemporaneous outcomes across three groups of participants: PEH aged 40 to 75 and those in stable housing 40 to 75 years for direct comparison; and PEH aged 18 to 39 years to acknowledge and explore the possibility of accelerated ageing measurable by these assessments. The outcomes of assessments administered to each group are also compared with expected normal values where these are available.

This comparison identifies the differing health needs of these groups living in the same city—Adelaide. Adelaide is the capital city of South Australia (SA) with 1.4 million inhabitants [15], where the number of PEH is reported as 5% (7428, up from 6224 in 2016)—slightly less than the national average [16]. This provides information to assist in the development of prioritised health interventions and services for PEH.

## 2. Materials and Methods

This cross-sectional, observational study compares the outcomes from a comprehensive set of health assessments with PEH in inner Adelaide, SA—Inspiring Health in the Inner City (IHIC)—with the outcomes of the same 16 health assessments collected in the same city, over the same 12-month period, for a group of non-homeless adults, dwelling in Adelaide—Inspiring Health (IH) [17]. Informed consent was obtained from all participants, and all methods were carried out in accordance with the relevant guidelines and regulations of Southern Adelaide Clinical Human Research Ethics Committee (SAC HREC). Ethics approval was provided from the SAC HREC: references 222.17 (IHIC) and 391.16 (IH).

### 2.1. Study Participants

The criteria for participating in IHIC were as follows: aged 18 years or older; ability to provide written consent; and homeless as per the Australian Institute of Health and Welfare (AIHW) definition of homelessness, that is, “if their current living arrangement: is in a dwelling that is inadequate; has no tenure, or if their initial tenure is short and not extendable, or does not allow them to have control of, and access to space for social relations” [13] (p. 94). To overcome the lack of a comprehensive register of PEH in this capital city, convenience sampling with snowballing was used [18]. Participants were invited by direct invitation from staff, or indirect invitation via peers, or other people known to them who had seen posters when they attended Common Ground or any of its service network sites. Data for IHIC were collected at the premises of a community housing project, Common Ground [19], which runs under the auspices of Housing Choices SA.

For IH, participants were aged 40 to 75 years, community dwelling in the greater Adelaide area, and able to provide written consent. Recruitment occurred via purposive sampling supported by extensive partnerships with local government and a national bank to attract people who would not normally seek out medical services for health screening or undergo a comprehensive health screening [17]. Data for IH were collected at local government and university venues.

### 2.2. Health Assessments

Before undertaking the health assessments, all consenting, eligible participants completed a standard physiological risk assessment to establish fitness for study participation and were excluded if pain was present or any screening physiological variables (e.g., temperature, heart, and respiratory rate) were outside of normal values. Feasibility studies and alterations in the ways these assessments were administered have been described elsewhere [19,20,21].

For all participants, assessments were delivered in sequential stations, staffed by appropriate health professionals (IHIC) or health students (IH) [22]. Data were collected in one 150 min session per person; participants moved around stations until all those they consented to had been completed. Appointments were available over six days for those at Common Ground (IHIC) and moved through council areas over a six-month period for the comparison group (IH), with participants able to choose a day and time within a two-week period for their preferred area. Data collection consisted of self-report using validated questionnaires and measurement by a qualified health professional; all assessments were informed by previous work [20,22]. At each station, the assessment was described in detail to the participant, and the participant was required to give verbal consent prior to participation. Participants could decline to participate in any assessment, and those considered at risk of harm from an assessment task were excluded from participating in that assessment but were able to proceed to others. Participants in IHIC were invited to a shared lunch either before, or after, their assessment and were given a selection of oral and personal hygiene products in acknowledgement of participation.

### 2.3. Data Collection and Management

The 16 assessments presented in this paper common to both groups and having validated population thresholds/norms are as follows: objective measurement of grip strength, lung function, and anthropometry (body mass index (BMI), waist circumference, waist-to-hip ratio, and muscle mass percentage); audiometry (Speech, Spatial and Qualities of Hearing Questionnaire (SSQ5)) [23,24]; cognition assessed as the General Practitioner Assessment of Cognition (GPCog) [25]; foot sensation (monofilament testing) [26]; frailty (Clinical Frailty Scale) [27]; self-report of falls/near falls in the past 6 months; psychological distress measured using the Kessler Psychological Distress Scale [K10]) [28]; sleep quality (Pittsburgh Sleep Quality Index (PSQI)) [24]; routine health screening (blood, bowel, pap, and prostate screens) in the past 12 months; pelvic floor bother; and visiting an emergency department (ED) in the past 12 months.

### 2.4. Data Analysis

Analyses were conducted using IBM SPSS Statistics version 29.0.2 (Clarivate, USA), R (version 4.4.1, R Foundation for Statistical Computing, Vienna, Austria), R studio [29], and Microsoft Excel (Microsoft 365 MSO, version 2407). Data are reported as means with SD for continuous data and percentages with confidence intervals (CIs) for categorical data using chi-squared (χ^2^) tests to ascertain statistical differences (95% CI; *p* value). Using published normative ranges or expected values, variables were recoded as ‘at risk’ where they lay outside of these ranges, or ‘not at risk’; gender–age group differences across groups were tested using χ^2^ models. The numbers of ‘at risk’ measures were then added together for each participant and summed to produce a deficit score. A comparison of the number of assessments where the participant is considered ‘at risk’ is made between the three age groups: group 1—PEH aged 18 to 39 years; group 2—PEH aged 40 to 75; and group 3—those in stable housing aged 40 to 75 years.

## 3. Results

In total, we had 612 participants who completed at least one of the 16 assessments: 15 from IHIC who were aged less than 40 years, 37 from IHIC aged 40 to 75 years, and 560 from IH (see Table 1). Samples differed by gender (*p* = 0.007) and age (*p* < 0.001); however, mean age was similar when comparing groups 2 and 3 (*p* = 0.168). Those experiencing homelessness were less likely to hold a diploma, undergraduate, or postgraduate degree or be married/de facto (*p* < 0.001).

There were many more differences than similarities in those failing to achieve a score within thresholds for health across the three groups, with PEH showing significantly poorer health outcomes across most assessments, when compared with those not experiencing homelessness in the same city (see Table 2). This was most extreme in the measures of clinical frailty, psychological distress, poor sleep quality, grip strength, lung function (spirometry), having fallen in the past six months, and bothersome pelvic floor health (all *p* < 0.001). Additionally, attendance at an emergency department in the last 12 months where no normative data are available also shows that PEH are significantly more likely to attend when compared with the IH cohort. Comparison across groups shows that those experiencing homelessness were also significantly less likely to be overweight (14.7%; *p* = 0.003) but significantly more likely to be obese (44.1%; *p* = 0.032), which is supported by the results of the waist-to-hip ratio where PEH were more likely to be at risk (77.4%, *p* = 0.018) when compared with the IH cohort (numbers were too small to present those who were underweight separately). There were also differences found for those having had a bowel, blood, pap, or prostate screening test, where all 53 (100.0%) PEH had been screened at Common Ground, compared with 52.1% of those living in the community (*p* < 0.001).

Table 3 shows that for those undertaking all of the 16 assessments (54%; n = 331/612), group one and group two were significantly more likely to be at risk for six or more (mean 6.5; SD 2.4; 73.3%; *p* = 0.031 and mean 8.7; SD 1.7) compared with those not experiencing homelessness (mean 5.0; SD 2.1; 40.2%).

## 4. Discussion

This study provides support to the growing body of evidence that PEH have poorer health status overall than people with stable housing across multiple performance domains and body systems [37,38]. With research showing that the mortality gap between these populations is due to conditions that can be effectively treated with appropriate and timely healthcare [39], this study provides evidence for priorities in preventative, health management service initiatives to support PEH. In this analysis, we show that PEH over the age of 40 years, being supported by a community housing project providing temporary housing, have worse health on a group of 16 health assessments when directly compared with a sample of seemingly healthy community-dwelling adults in stable housing of the same age. However, what we add to the knowledge base is that PEH of younger ages show clear evidence of accelerated ageing as they do not show better health, but in fact show just as many deficits as PEH over the age of 40 years. We identified health deficits and poor performance for more than 20% of PEH in both age groups in 14 out of the 16 health assessments reported here (87.5%), with 10 (62.5%) of these showing statistically significantly worse health outcomes for PEH compared with those people with stable housing, in the same Australian capital city, where only eight are identified (50.0%). This disparity indicates the increased need for prevention and health management services specific to the needs of PEH, if comparable health status is to be achieved, and highlights the overall poorer health of PEH compared with those in safe, more permanent housing [8].

Poorer outcomes for behavioural indicators associated with privacy, self-care, and maintaining good health, such as sleep quality and psychological distress, have been reported by PEH to be inevitable due to their environmental contexts [9]. A study of 2144 healthy subjects aged between 43 and 71 years in Spain found that approximately 40% reported poor sleep quality using the same instrument (PSQI) [40], twice what we found here in the Adelaide stable housing cohort (22.7%) using the same instrument; however, this increased to nearly half (48.5%) of the cohort of PEH of similar age and three and half times (78.6%) for younger PEH. Poor sleep quality has been reported in approximately four in five people with poor mental health [41], and findings from our study concur with a strong relationship between homelessness, poor-quality sleep, and poor mental health. Recent results from the Australian National Study of Mental Health and Wellbeing, 2020-21 [42] show that 15% of adults experience high or very high levels of psychological distress, with the most economically disadvantaged populations reporting distress rates at much higher than the national average (25%) [43]. These rates are echoed in this study among the stable housing cohort (16.6%) but are still much lower than the proportion of PEH reporting elevated psychological distress (48.5%) of the same age reported here, rising to 71.4% of those aged less than 40 years (group 1).

In addition to the high levels of psychological distress, nearly one third of PEH in group two returned below-threshold scores in cognitive assessments compared with the proportion among people in stable housing (less than 20%). This finding is supported by an umbrella review of cognitive functioning in homeless adults, which reported studies that found that up to 55% of PEH were cognitively deficient [44]. In the Australian population, the prevalence of cognitive impairment is currently unknown, but an international review estimated the standardised prevalence of mild cognitive impairment in adults aged 60 years and older to be between 6% and 12% [45], far lower than in both populations compared here. This may indicate an increase in mild cognitive impairment generally, or this may in part be attributed to the different tools used and applied criteria for mild cognitive decline. Another indicator of accelerated ageing is frailty, and homelessness is known to be associated with accelerated ageing and incipient frailty [46,47]. A study undertaken in the same Australian state recently found that frailty rates were between 9% and 12% in the general population [48]. Here, frailty was identified in just under 7.0% of participants aged between 40 and 75 years in the stable housing group; however, it was four and half times higher among PEH of the same age (44.4%). This highlights the attributes of frailty and accelerated ageing in this cohort, supporting other research, and suggesting that homelessness is associated with increased frailty [49].

As in other studies, it was found here that the numbers of those with unhealthy weight were comparable between cohorts (slightly lower for group 2 and higher for group 1, but not significantly). Koh and colleagues attribute the similar proportions of overweight/obese people in community dwelling and homeless populations in Boston (USA) to the hunger–obesity paradox, where poor individuals eat cheap, energy-dense foods resulting in the co-existence of hunger and obesity [50]. The Boston cohort reported a much lower prevalence of underweight people (1.6%) than the PEH in this sample (13.1%), which cannot be explained. The mechanisms between food insecurity and BMI are complex, and there is limited literature examining these phenomena in homeless populations [50]. However, we do know that a healthy diet reflects appropriate intake of nutrients and protects against chronic disease, poor general health, and premature ageing [51,52], which are all challenges for PEH. This is complicated by the evidence of crisis management of health with the high number of visits to an emergency department in the past twelve months (11.3% among IH rising to over 30% among the PEH groups). However, the 100% uptake of medical screening activities by PEH indicates that when the services are affordable and provided in a familiar and ‘safe’ setting, there is willingness to participate, and that health literacy seems to underpin self-management practices. This is a positive indicator for the development of and likely participation of PEH in expanded health services delivered in this type of environment and reinforces the recommendation for these venues and services in health and social policy [11].

On further investigation, we also found evidence that health disparities are specific to the age groups within the PEH populations. This is demonstrated by the lower number of health deficits in PEH aged 40–75 years compared with those less than 40 years, with the younger age group experiencing significantly more deficit areas. Younger PEH have poor health status related to distress and poor sleep quality compared with PEH 40 years or over who show poorer health status that may be more related to the expression of chronic health conditions (e.g., waist circumference and waist-to-hip ratio, airflow limitation in the lungs, poor foot sensation, cognition, and clinical frailty). These findings support our recommendations for expanded services for PEH and stress the importance of health and social policy addressing a difference within the PEH population as well as between PEH and those in stable housing.

The authors acknowledge several limitations in this study predominantly due to selection bias and the small sample, especially of PEH aged 40 to 75 years. It was not possible to find a reference population for those PEH in inner city Adelaide, and those able to be contacted were done so with the assistance of Common Ground. Both cohorts were recruited using convenience sampling with snowballing—assisted by local councils and a national bank for the stable housing cohort and Common Ground and their networks for PEH. It is acknowledged that these methods reflect only populations known to the staff of these organisations and associated networks and, therefore, had reached only a limited sample of all PEH and stable housing. Due to this, we opened the age range up to all adults (18 years and above) to ensure that we found the largest number of PEH willing to participate. We acknowledge that the difference in the inclusion criteria for IH and IHIC is a limitation in these analyses; however, we attempted to minimise this limitation by presenting PEH 40 to 75 years of age separately to PEH less than 40 years in these analyses. It is further acknowledged that this may have impacted the ‘volunteering’ nature of our selection due to the possibility of coercion due to power imbalance between those responsible for recruitment and participants, especially in the cohort of PEH. This may have had consequences for participant rates. However, a strength of this study is that it compared data captured using the same assessment instruments, measured in the same way in the same period, from similarly aged people, in the same city, with samples differentiated only by their accommodation. The assessments captured information on the performance of potentially inter-related body systems, comprising musculoskeletal (anthropometry, grip strength); central neural function (cognition, hearing); respiratory function (lung ratio); and mental health (sleep quality, psychological distress). Although the sample size is small overall, it is not too small to make statistical inferences. However, where CIs are wide, results should be interpreted with care. Due to the strict measurement criteria, the similarities in data collection methods, and the restriction of age to 40–75 years for direct analyses between groups two and three, we believe that we have a large-enough body of evidence to show the impact of homelessness. However, we note that generalisations outside of the context of an Australian capital city setting should be made with caution.

Added to these considerations is the potential for over- or under-estimation of some data due to the self-report collection methods where participants may give answers that they see as appropriate to the purpose of study. In addition, participants in the cohort of PEH may have been familiar with testing measures such as the K10 instrument [33], and this may have led to latent Hawthorne effects due to the participant knowing that they were being observed [53]. However, the reasons for these consistently significantly poorer findings for the PEH more likely reflect the reality of homelessness and its contexts in a large city. There is increasing evidence that homelessness compromises physical and mental health and is associated with premature ageing [37]. Thus, compared with similarly aged people in the general population, PEH are likely to suffer earlier and more age-related chronic health conditions [38]. Chronic age-related health conditions can include body systems failure (cardiac, respiratory, musculoskeletal, or renal conditions); cognitive impairment; functional decline; and frailty [54,55]. Cumulatively, such chronic conditions increase the mortality and morbidity of PEH [56]. The poor sleep quality, poorer mental health, and poorer cognition and memory found in this cohort may reflect the lack of safety/security in lived environments (for instance, shared accommodation), unstructured daily routines, and/or not being in employment [57,58]. Being at risk for obesity, waist circumference, and waist-to-hip ratio may reflect a lack of finances for healthy food, irregular meals, and food insecurity in terms of lack of opportunity for meal preparation and safe food storage [58].

## 5. Conclusions

The information in this paper highlights just how poor the health of PEH is in an Australian capital city and how the lack of housing security impacts mental and physical health. Homelessness is a complex but potentially reversible social circumstance, and the differences in health needs between those in stable housing and those who are not indicate the need for targeted health assessments and service provision for PEH. While adding to the growing body of evidence that PEH have poorer health status overall than people in stable housing, across multiple performance domains and body systems [37,38], this study shows how a relatively simple group of routine assessments can be used to identify the significant health deficits among PEH that are likely to contribute to accelerated ageing. It provides evidence for targeted initiatives to support PEH to provide the greatest impact in addressing health inequalities.

## Figures and Tables

**Table 1:** Demographic characteristics: comparison of PEH less than 40 years, PEH 40 to 75 years, and participants 40 to 75 years who were in secure housing, over the same period, in the same capital city.

	*p* Value	Grp.1 IHIC 18–39 yrs	Grp.2 IHIC 40–75 yrs	Grp.3 IH 40–75 yrs	Total 18–75 yrs
**Age in years (m, SD)**	<0.001 *	31.1 (6.8)	55.5 (9.8)	60.2 (10.2)	59.2 (11.1)
		n (%, CI)	n (%, CI)	n (%, CI)	n (%, CI)
**Gender**	0.007 *				
Male		**9 (60.0, 32.3–83.7)**	**20 (54.1, 36.9–70.5)**	182 (32.5, 28.6–36.5)	211 (34.5, 30.7–38.4)
Female		6 (40.0, 16.3–67.7)	17 (45.9, 29.5–63.1)	**378 (67.5, 63.4–71.4)**	**401 (65.5, 61.6–69.3)**
**Level of qualifications**	<0.001 *				
High school/certificate/trade		**15 (100.0, 78.2–100.0)**	**35 (100.0, 90.1–100.0)**	289 (52.5, 48.3–56.8)	339 (56.5, 52.4–60.5)
Diploma/degree		0 (0.00, 0.00–21.8)	0 (0.00, 0.00–10.0)	**261 (47.5, 43.2–51.7)**	**261 (43.5, 39.5–47.6)**
**Primary language spoken**	0.807				
English		14 (93.3, 68.0–99.8)	32 (91.4, 76.9–98.2)	531 (94.8, 92.6–96.5)	577 (94.6, 92.5–96.2)
Language other than English		1 (6.7, 0.1–31.9)	3 (8.6, 18.0–23.0)	29 (5.2, 34.9–73.5)	33 (5.4, 3.7–7.5)
**Marital status**	<0.001 *	(n = 15)			
Single		**13 (86.7, 59.3–98.3)**	**21 (58.3, 40.7–74.5)**	61 (11.1, 7.0–12.0)	95 (15.8, 13.0–19.0)
Married/de facto		2 (13.3, 1.6–40.5)	10 (27.8, 14.2–45.2)	**367 (66.7, 62.6–70.6)**	**379 (63.1, 59.1–66.9)**
Divorced/separated/widowed		0 (0.00, 0.00–21.8)	5 (13.9, 4.7–29.5)	122 (22.2, 18.6–25.7)	127 (21.1, 17.9–24.6)

* Statistically significant difference between groups; grp: group; m: mean; n: number observed; SD: standard deviation; yrs: years.

**Table 2:** Age and gender adjusted percentages of PEH (18 to 39 years; 40 to 75 years) and participants who were in secure housing (40 to 75 years), compared with expected normal values where available, over the same period in the same capital city (normative data given for reference).

	*p* Value	IHIC (18–39 yrs) n (%, CI)	IHIC (40–75 yrs) n (%, CI)	IH (40–75 yrs) n (%, CI)	Total (18–75 yrs) n (%, CI)	Established/Expected Healthy Ranges
**Anthropometry [30]**						
Unhealthy weight	0.537	10 (71.4, 41.9–91.6)	20 (58.8, 40.7–75.3)	372 (67.6, 63.5–71.5)	402 (67.2, 63.3–80.0)	18–25 kg/m^2^
Waist circumference	0.512	9 (64.3, 35.1–87.2)	21 (67.7, 45.1–79.6)	415 (74.4, 70.5–77.9)	445 (73.8, 70.1–77.3)	Male < 94 cm; female < 80 cm
Waist-to-hip ratio	0.061 ^‡^*	**8 (57.1, 28.9–82.3)**	**24 (77.4, 58.9–90.4)**	312 (55.8, 51.6–60.0)	344 (57.0, 52.9–60.9)	Male ≤ 90%; female ≤ 85%
Low muscle mass	0.396	10 (71.4, 41.9–91.6)	17 (50.0, 32.4–67.5)	315 (56.3, 52.0–60.4)	342 (56.3, 52.2–47.8)	Male (41–60 yr) 33.2–39.2%; (61–80 yr) 33.0–38.7% Female (41–60 yr) 24.2–30.3%; (61–80 yr) 24.0–29.8%
**Audiometry [23]**						
Uni- or bi-lateral hearing loss	0.921	4 (26.7, 7.9–55.1)	10 (31.3, 16.1–50.0)	170 (31.6 (27.7–35.7)	184 (31.5, 27.7–35.4)	SSQ5 > 7.3
**Foot sensation [26]**						
Monofilament test (score < 10)	0.248	0 (0.00, 0.0–24.7)	5 (16.1, 5.4–33.7)	53 (9.8, 7.4–12.6)	58 (9.9, 7.6–12.6)	All 10 sites intact
**Lung function (spirometry) [31]**						
Any airflow limitation	<0.001 *	**5 (33.3, 11.8–61.6)**	**19 (59.4, 406–76.3)**	83 (17.4, 14.1–21.1)	107 (20.4, 17.0–24.1)	No airflow limitation
**Grip strength (dynamometer; kg) [32]**						
Weaker than age and gender norms	<0.001 *	**2 (18.2, 2.3–51.8)**	**9 (28.1, 13.7–46.7)**	30 (7.2, 4.9–10.2)	41 (9.0, 6.5–12.0)	Male (40–49 yr) 37.5–56.5; (50–59 yr) 36.6–53.4; (60–69 yr) 31.7–48.3; (70+ yr) 25.2–40.8 female (40–49 yr) 23.3–34.7; (50–59 yr) 21.7–34.3; (60–69 yr) 18.7–29.3; (70+ yr) 14.2–25.8
**Cognition (GP Cog) [25]**						
Impaired cognition	0.134	4 (28.6, 8.4–58.1)	10 (31.3, 16.1–50.0)	101 (18.4, 15.2–21.8)	115 (19.3, 16.2–22.7)	8/9 or 9/9
**Clinical Frailty Scale [27]**						
Vulnerable, mild-moderate frailty	<0.001 *	**6 (40.0, 16.3–67.7)**	**16 (44.4, 27.9–61.9)**	37 (6.8, 4.8–9.3)	59 (9.9, 7.6–12.6)	Score of 1–3/9
**Psychological distress (K10) [33,34]**						
High to very high (score ≥ 22)	<0.001 *	**10 (71.4, 41.9–91.6)**	**16 (50.0, 31.9–68.1)**	93 (16.6, 13.6–19.9)	119 (19.6, 16.5–23.0)	K10 score < 22
**Pelvic floor health–overall [35]**						
Some or severe bother	<0.001 *	**14 (100.0, 76.8–100.0)**	**26 (100.0 (86.8–100.0)**	359 (72.4, 68.2–76.3)	399 (74.4, 70.5–78.1)	Score of 0 on all nine domains
**Sleep Quality (PSQI)** [36]						
Poor sleep quality (score 8–21)	<0.001 *	**11 (78.6, 49.2–95.3)**	**16 (48.5, 30.8–66.4)**	110 (22.7, 19.0–26.7)	137 (25.8, 22.1–29.7)	PSQI score of 0–7 points
**Falls/near falls**						
At least one in the last 6 months	0.001 *	**4 (26.7, 7.9–55.1)**	**17 (48.6, 31.4–66.0)**	82 (14.7, 11.9–18.0)	103 (17.0, 14.1–20.2)	None
**Visit to emergency department**						
In the last 12 months	<0.001 *	**5 (33.3, 11.8–61.6)**	**12 (32.4, 18.0–49.8)**	63 (11.3, 8.7–14.2)	80 (13.1, 10.5–16.0)	None
**Not having undertaken screening**						
Blood, bowel, pap, or prostate	<0.001 *	0 (0.0, 0.0–21.8)	0 (0.0, 0.00–9.5)	**292 (52.1, 47.9–56.3)**	292 (47.7, 43.7–51.7)	At least one screening

^‡^ *p* values approaching 0.05 are not always a reliable measure of significance (https://www.ncbi.nlm.nih.gov/pmc/articles/PMC6532382/ (accessed on 14 August 2024)). Proportions, CIs, and *p* values are shown to indicate differences between the two groups. These are denoted by an asterisk (*). Where CIs are wide, results should be interpreted with care. BMI: body mass index; cm: centimetres; ED: emergency department; GPCog: General Practitioner Assessment of Cognition; IH: Inspiring Health (community dwelling in secure housing); IHIC: Inspiring Health in the Inner City (people experiencing homelessness); K10: Kessler Psychological Distress Scale (10); kg: kilogram; n: number observed; PSQI: Pittsburgh Sleep Quality Index; SSQ: Speech, spatial and qualities of hearing; yr: years.

**Table 3:** Number of at-risk assessments per participant for the 16 assessments (n = 331)—PEH compared with participants who were in secure housing, over the same period in the same capital city.

Deficits	IHIC Participants (18–39 yrs; n = 8)	IHIC Participants (40–75 yrs; n = 22)	IH Participants (40–75 yrs; n = 301)	Total Participants (18–75 yrs; n = 331)
Mean (SD)	8.7 (1.7)	6.5 (2.4)	5.0 (2.1)	5.2 (2.2)
	**n (%, CI)**	**n (%, CI)**	**n (%, CI)**	**n (%, CI)**
5 or less *	0 (0.00–36.9)	8 (36.4, 17.2–59.3)	**180 (59.8, 54.0–65.4)**	188 (58.2, 52.6–63.6)
6 or more *	**8 (100.0, 63.0–100.0)**	**14 (63.6, 40.6–82.8)**	121 (40.2, 34.6–50.0)	135 (41.8, 36.3–47.4)
6 to 8	nr	8 (36.4, 17.2–59.3)	105 (34.9, 29.5–40.6)	113 (35.3, 29.8–40.4)
9 or more *	nr	**6 (27.3, 10.7–50.2)**	16 (5.3, 3.1–8.5)	22 (6.8, 4.3–10.1)

* *p* < 0.001; 95% CIs not overlapping (IHIC compared with IH)—bold showing statistically more likely. CI: confidence interval; IH: Inspiring Health (community dwelling in secure housing); IHIC: Inspiring Health in the Inner City (people experiencing homelessness); n: number observed; nr: not reported due to small numbers; SD: standard deviation; yrs: years.

## Data Availability

Datasets used and/or analysed during the current study are available from the corresponding author on reasonable request.
